# Phenotypic and genotypic characterization of carbapenem-resistant *Acinetobacter baumannii* isolates from Egypt

**DOI:** 10.1186/s13756-019-0611-6

**Published:** 2019-11-20

**Authors:** Alaa Abouelfetouh, Aisha S. Torky, Elsayed Aboulmagd

**Affiliations:** Department of Microbiology and Immunology, Faculty of Pharmacy, Alexandria University, 1 Khartoum Sq., Azarita, Alexandria, 21521 Egypt

**Keywords:** *Acinetobacter baumannii*, Carbapenemase prevalence, CarbAcineto NP test, *bla*_*OXA-51*_, *bla*_*OXA-23*_, *bla*_*NDM*_

## Abstract

**Background:**

Antibiotic use is largely under-regulated in Egypt leading to the emergence of resistant isolates. Carbapenems are last resort agents to treat *Acinetobacter baumannii* infections resistant to other classes of antibiotics. However, carbapenem-resistant isolates are emerging at an alarming rate. This study aimed at phenotypically and molecularly characterizing seventy four carbapenem-unsusceptible *A. baumannii* isolates from Egypt to detect the different enzymes responsible for carbapenem resistance.

**Methods:**

Carbapenemase production was assessed by a number of phenotypic methods: modified Hodge test (MHT), carbapenem inactivation method (CIM), combined disc test (CDT), CarbAcineto NP test and boronic acid disc test. Polymerase chain reaction (PCR) was used to screen the isolates for the presence of some genes responsible for resistance to carbapenems, as well as some insertion sequences.

**Results:**

PCR amplification of class D carbapenemases revealed the prevalence of *bla*_*OXA-51*_ and *bla*_*OXA-23*_ in 100% of the isolates and of *bla*_*OXA-58*_ in only one isolate (1.4%). *bla*_*VIM*_ and *bla*_*NDM-1*_ belonging to class B metallo-β-lactamases were present in 100 and 12.1% of the isolates, respectively. The prevalence of IS*Aba1*, IS*Aba2* and IS*Aba3* was 100, 2.7 and 4.1%, respectively. None of the tested isolates carried *bla*_*OXA-40*_, *bla*_*IMP*_, *bla*_*SIM*_, *bla*_*SPM*_, *bla*_*GIM*_ or the class A *bla*_*KPC*_. Taking PCR as the gold standard method for the detection of different carbapenemases, the sensitivities of the MHT, CIM, CDT, CarbAcineto NP test and boronic acid disc/imipenem or meropenem test for this particular collection of isolates were 78.4, 68.9, 79.7, 95.9, and 56.8% or 70.3%, respectively.

**Conclusions:**

The widespread detection of carbapenem-resistant *A. baumannii* (CR-AB) has become a real threat to the efficacy of treatment regimens. Among the studied cohort of CR-AB clinical isolates, *bla*_*OXA-51*_, *bla*_*OXA-23*_ and *bla*_*VIM*_ were the most prevalent, followed by *bla*_*NDM-1*_ and *bla*_*OXA-58*_. The genotypic detection of carbapenemases among CR-AB clinical isolates using PCR was most conclusive, followed closely by the phenotypic testing using CarbAcineto NP test.

## Background

*Acinetobacter baumannii* has become a life threatening pathogen [1]. It causes nosocomial infections worldwide, including skin and soft tissue infections, wound and bloodstream infections, urinary tract infections, meningitis and ventilator-associated pneumonia which is the most common and fatal infection caused by *A. baumannii* [2–6]. These infections are particularly dangerous because of the pathogen’s ability to resist the action of most currently available antibacterial agents [7], making *A. baumannii* one of the most dangerous ESKAPE organisms [*Enterococcus faecium*, *Staphylococcus aureus*, *Klebsiella pneumoniae*, *A. baumannii*, *Pseudomonas aeruginosa* and *Enterobacter* species] [8, 9]. Moreover, the number of community-acquired *A. baumannii* infections such as bacteraemia, pneumonia, meningitis and endocarditis has been progressively increasing in the last few decades [10, 11].

The unnecessary and extensive use of antibiotics in healthcare settings led to the acquisition of novel genetic determinants for antibiotic resistance. These, added to the intrinsic resistance of *A. baumannii* to many antibiotics, resulted in the development of multidrug-resistant, extensively drug-resistant and even pan drug-resistant *A. baumannii* strains, mainly in intensive care settings [12–14], limiting the options available to treat such infections to a few agents such as the carbapenems [15]. Yet, *A. baumannii* has also developed resistance to carbapenems and carbapenem-resistant *A. baumannii* (CR-AB) isolates have been reported all over the world, challenging modern day medicine [16].

Carbapenem resistance among *A. baumannii* strains occurs due to the loss or modification of porins or in some rare cases due to modification of penicillin binding proteins [17]. However, the main resistance mechanism is the production of β-lactamase enzymes [18–20]. Four molecular β-lactamase classes (A, B, C and D) have been detected in *A. baumannii* [21, 22]. Only a few *K. pneumoniae* carbapenemase (KPC) type enzymes from class A β-lactamases have an effect on carbapenems contrary to classes B and D which act efficiently on carbapenems [23]. Class B β-lactamases are metallo-β-lactamases (MBL) that need zinc ions for their catalytic activity [23]. Four examples of MBL are known in *A. baumannii*, including New Delhi Metallo-β-lactamase (NDM), Imipenemase (IMP), Seoul Imipenemase (SIM) and Verona integron-encoded metallo-β-lactamase (VIM) [24, 25]. Two variants of NDM have been reported (NDM-1 and NDM-2). The two variants have been reported in Egyptian *A. baumannii* clinical isolates. The first variant (NDM-1) was detected in a repatriated Czech citizen after being admitted to a hospital in Egypt in 2011, it is not clear whether the patient was colonized or infected [26]. The second variant (NDM-2) was isolated in Germany from a venous line catheter placed for a child while being hospitalized in Egypt [27]. Regarding Class D β-lactamases, also named oxacillinases (OXAs) for their activity on oxacillin [28], there are six subgroups: the naturally occurring, chromosomal and intrinsic OXA-51 and the acquired OXA-23-like, OXA-58-like, OXA-24/40-like, OXA-235-like and OXA-143-like β-lactamases [29]. The presence of certain insertion sequences upstream of carbapenem-hydrolyzing class D β-lactamase (CHDLs) genes leads to their over-expression, conferring carbapenem resistance [30].

Detection of the carbapenemases is crucial to determine the severity of the problem and to direct the application of antimicrobial stewardship guidelines to limit further evolution of carbapenem-resistant variants among *A. baumannii* isolates. The current study is reporting the prevalence of certain carbapenemases among CR-AB isolates from Alexandria, Egypt, comparing the different phenotypic and molecular techniques to detect these enzymes among CR-AB isolates in an Egyptian setting.

## Methods

### Bacterial isolates

Seventy four CR-AB isolates, from different clinical specimens: broncho-alveolar lavage (BAL) (*n* = 43), urine (*n* = 7), blood (*n* = 6), sputum (*n* = 6), pus (*n* = 4), miniBAL (*n* = 4), wound (*n* = 2) and tissue (*n* = 2), collected from the medical microbiology lab at Alexandria Main University Hospital in 2010 (*n* = 39) and 2015 (*n* = 35) were included in the study. The isolates were mostly collected during fall and winter 2010 and spring 2015, were non-duplicate and came from different wards in the hospital. The isolates were identified by phenotypic and growth based methods, including colony morphology, aerobic growth on MacConkey’s agar at 44 °C [31] and Vitek system (Biomerieux, UK). The identity was confirmed by molecular methods, namely the polymerase chain reaction (PCR) amplification of *bla*_*OXA-51*_ gene [32] as well as Matrix Assisted Laser Desorption Ionization - Time of Flight Mass Spectrometry (MALDI-TOF MS) (Bruker Daltonik, USA) [33, 34].

Carbapenem susceptibility of the clinical isolates was checked by the standard disc diffusion technique on Müller-Hinton agar using imipenem and meropenem discs (Oxoid, Basingstoke, United Kingdom), according to CLSI 2018 guidelines [35]. The minimum inhibitory concentration (MIC) of imipenem (Merck Sharp & Dohme B.V., The Netherlands) and meropenem (Astrazeneca, United Kingdom) were determined against the tested clinical isolates using agar dilution method to confirm carbapenem resistance, and the results were interpreted according to CLSI 2018 guidelines (data not shown) [35].

### Phenotypic detection of carbapenemases

#### Modified Hodge test (MHT)

MHT was performed as descried before [36]. One to ten dilution of 0.5 McFarland suspension of the carbapenem-susceptible *E. coli* ATCC 8739 was aseptically swabbed onto a sterile Müller-Hinton agar plate. A meropenem disc (10 μg) was aseptically placed in the center of the plate. In a straight line from the interior to the exterior of the plate, each tested isolate was streaked. *K. pneumoniae* ATCC 10031 was used as a negative control. The plates were then incubated for 18–24 h at 37 °C then examined for a clover leaf-type indentation in the inhibition zone of the carbapenem disc at the intersection of the test organism and *E. coli* ATCC 8739.

#### Carbapenem inactivation method (CIM)

CIM test was performed as previously described [37], with some modifications. Meropenem disc (10 μg) was incubated for 4 h in an overnight culture of the tested bacterial isolates. A 0.5 McFarland suspension of *E. coli* ATCC 8739 was swabbed onto Müller-Hinton agar. After incubation, the meropenem disc was placed onto the inoculated Müller-Hinton agar plate and incubated for 18–24 h at 37 °C. The presence of a clear inhibition zone (≥ 20 mm) indicated the absence of carbapenemase activity.

#### Combined disc test (CDT)

On a Müller-Hinton agar plate inoculated with 1:10 dilution of 0.5 McFarland suspension of *E. coli* ATCC 8739, imipenem (10 μg) and imipenem/EDTA (10/930 μg) discs (Oxoid, Basingstoke, United Kingdom) were placed, at a distance of no less than 20 mm between the centers of the discs. After 18–24 h of incubation at 37 °C, the diameters of the inhibition zones around the discs were compared [38]. An increased inhibition zone of ≥ 7 mm with the imipenem/EDTA disc compared to the imipenem disc alone was considered an indication for MBL production.

#### CarbAcineto NP test

Two to three colonies of each tested isolate growing on Luria-Bertani (LB) agar plate were picked up and suspended in two Eppendorf tubes (A and B) containing 100 μL of 5 M NaCl. Both tubes A and B also contained 100 μL of revealing solution in addition to 6 mg/mL imipenem in tube B. The revealing solution comprised of phenol red as pH indicator and 0.1 mmol/L ZnSO_4_. The phenol red solution was prepared by adding 2 mL of a phenol red solution 0.5% (wt/vol) to 16.6 mL of distilled water and then adjusting the pH value to 7.8 by adding 1 N NaOH. After a maximum incubation time of 2 h at 37 °C, tubes A and B were visually inspected for color change. In tube B, the carbapenemase activity was detected by a color change of phenol red solution (red to yellow/orange) resulting from the hydrolysis of imipenem into a carboxylic derivative, leading to a decrease of the pH value [39].

#### Boronic acid disc test

Ten microliters of 3-aminophenylboronic acid (PBA) dissolved in dimethyl sulfoxide (DMSO) (40 mg/mL) equivalent to 400 μg PBA solution were aseptically dropped onto imipenem and meropenem discs. Treated and untreated imipenem and meropenem discs were also transferred onto Müller-Hinton agar plate inoculated with the tested isolate. In addition, 400 μg PBA disc lacking either antibiotic was used on the same plate as a control. After incubation for 18–24 h at 37 °C, the inhibition zone diameters around the treated imipenem and meropenem discs were compared with the diameters around the plain antibiotic discs. A ≥ 5 mm difference in zone diameter was considered as a positive result [40].

### Molecular characterization of resistance determinants

For preparation of DNA template, four colonies of each tested clinical isolate were suspended in 200 μL sterile deionized water. The suspension was heated at 95 °C for 30 min and then frozen at − 20 °C for 30 min. After thawing, the tube was centrifuged at 14,000 rpm for 10 min. The supernatant was then aliquoted and preserved at − 20 °C for future use [32]. The presence of carbapenemase genes belonging to class A (*bla*_*KPC*_), B (*bla*_*IMP*_*, bla*_*VIM*_*, bla*_*SIM*_*, bla*_*SPM*_*, bla*_*GIM*_ and *bla*_*NDM*_) and D (*bla*_*oxa-23*,_ *bla*_*oxa-40*,_ *bla*_*oxa-51* and_ *bla*_*oxa-58*_) and insertion sequences: IS*Aba1*, IS*Aba2* and IS*Aba3* was investigated in the extracted DNA using PCR, following the conditions detailed in Table 1. All primers are listed in Table 2. Both strands of the NDM amplicons were sequenced using an ABI 3500XL genetic analyzer (Inqaba Biotechnologies, Pretoria, South Africa).

**Table 1:** PCR conditions of amplification

Carbapenemase class/Insertion sequence	Gene	Thermal cycling conditions					Master-mix used
		Initial denaturation	30 cycles			Final extension	
			Denaturation	Annealing	Extension		
Class D carbapenemases [41]	*bla*_*oxa-51*_ *bla*_*oxa-23*_ *bla*_*oxa-58*_ *bla*_*oxa-40*_	95 °C /1 min	95 °C /15 s	52 °C /15 s	72 °C /10 s	72 °C /10 min	MyTaq™ HS Mix
Class B carbapenemases [42, 43]	*bla*_*IMP*_	94 °C /5 min	94 °C /40 s	47 °C /1 min	72 °C /2 min	72 °C /5 min	OnePCR™
	*bla*_*VIM*_	94 °C /5 min	94 °C /40 s	51 °C /1 min			
	*bla*_*SIM*_ *bla*_*SPM*_ *bla*_*GIM*_	94 °C /5 min	94 °C /40 s	52 °C /1 min			
	*bla*_*NDM*_	94 °C /5 min	94 °C /40 s	54 °C /1 min			
Class A carbapenemases [44]	*bla*_*KPC*_	94 °C /5 min	94 °C /40 s	54 °C /1 min	72 °C /2 min	72 °C /5 min	OnePCR™
Insertion sequences [17]	IS*Aba1* IS*Aba2* IS*Aba3*

**Table 2:** Oligonucleotide primers sequence and amplicon size

Primer name	Nucleotide sequence (5′ → 3′)	No. of bases	Size of the amplicons (bps)
OXA-51-F [41]	TAATGCTTTGATCGGCCTTG	20	353
OXA-51-R [41]	TGGATTGCACTTCATCTTGG	20	
OXA-23-F [41]	GATCGGATTGGAGAACCAGA	20	501
OXA-23-R [41]	ATTCTTGACCGCATTTCCAT	20	
OXA-40-F [41]	GGTTGTTGGCCCCCTTAAA	19	246
OXA-40-R [41]	AGTTGAGCGAAAAGGGGATT	20	
OXA-58-F [41]	AAGTATTGGGGCTTGTGCTG	20	599
OXA-58-R [41]	CCCCTCTGCGCTCTACATAC	20	
IS*Aba1*-F [17]	GTGCTTTGCGCTCATCATGC	20	430
IS*Aba1*-R [17]	CATGTAAACCAATGCTCACC	20	
IS*Aba2*-F [17]	AATCCGAGATAGAGCGGTTC	20	1100
IS*Aba2*-R [17]	TGACACATAACCTAGTGCAC	20	
IS*Aba3*-F [17]	CAATCAAATGTCCAACCTGC	20	403
IS*Aba3*-R [17]	CGTTTACCCCAAACATAAGC	20	
Pre NDM-F [42]	CACCTCATGTTTGAATTCGCC	21	984
Pre NDM-R [42]	CTCTGTCACATCGAAATCGC	20	
Imp-F [43]	GGAATAGAGTGGCTTAAYTCTC	22	188
Imp-R [43]	CCAAACYACTASGTTATCT	19	
Vim-F [43]	GATGGTGTTTGGTCGCATA	19	390
Vim-R [43]	CGAATGCGCAGCACCAG	17	
Gim-F [43]	TCGACACACCTTGGTCTGAA	20	477
Gim-R [43]	AACTTCCAACTTTGCCATGC	20	
Spm-F [43]	AAAATCTGGGTACGCAAACG	20	271
Spm-R [43]	ACATTATCCGCTGGAACAGG	20	
Sim-F [43]	TACAAGGGATTCGGCATCG	19	570
Sim-R [43]	TAATGGCCTGTTCCCATGTG	20	
KPC-F [44]	GTATCGCCGTCTAGTTCTGC	20	637
KPC-R [44]	GGTCGTGTTTCCCTTTAGCC	20	

## Results

### Phenotypic detection of carbapenemases

Seventy four carbapenem-resistant isolates were screened for carbapenemase production by a number of phenotypic methods (MHT, CIM, CDT, CarbAcineto NP test and boronic acid disc test) (Table 3). Regarding MHT, 58 isolates (78.4%) showed positive result, while the test failed to detect carbapenemase production in the remaining sixteen isolates (21.6%). Concerning the CIM, 51 isolates (68.9%) were carbapenemase producers. Fifty nine of the isolates (79.7%) were CDT positive, whereas 42 (56.8%) and 52 (70.3%) isolates showed positive boronic acid disc test in combination with imipenem and meropenem, respectively. CarbAcineto NP test developed positive results with 71 (95.94%) of the tested isolates. Four isolates (5.4%) developed the positive result displayed as color change from red to yellow in < 15 min. On the other hand, 67 isolates (90.5%) turned positive after 2 h of incubation. Only two isolates (2.7%) developed non-interpretable results and one isolate (1.4%) produced a negative result. However, only 21 isolates (24.3%) displayed positive results in all phenotypic tests. It is noteworthy though that at least one of the phenotypic tests was capable of detecting carbapenemase presence in all tested isolates.

**Table 3:** Detection of carbapenemases, their encoding genes and insertion sequences among the *A. baumannii* isolates

Code of isolates	No. of isolates	Test result for						Carbapenemase genes and insertion sequences
		MHT	CIM	CDT	CarbAcineto NP	Boronic acid disc		
						Imipenem	Meropenem	
A4a, A8a, A10a, A6135a, A14, A27, A41, A71, A72, A73, A76, A80, A83, A84, A88	15	+	+	+	+	+	+	*bla*_*OXA-51*_, *bla*_*OXA-23*_, *bla*_*VIM*_, IS*Aba1*
A6, A8, A12, A46, A69	5	+	+	+	+	–	+	
A1a, A14a, A24, A36	4	+	–	+	+	–	+	
A11a, A25, A37, A68, A91	5	+	+	+	+	–	–	
A30, A34, A64, A74	4	–	–	+	+	+	+	
A42, A43, A92	3	+	–	+	+	+	+	
A15, A16, A82	3	+	+	–	+	–	–	
A13, A22	2	+	+	–	+	+	+	
A4, A31	2	–	+	+	+	–	+	
A7, A39	2	–	+	+	+	+	–	
A10, A78	2	+	–	+	+	–	–	
A90	1	+	+	+	+	+	–	
A5	1	–	+	+	+	+	+	
A18	1	+	–	–	+	+	+	
A45	1	+	–	–	+	+	–	
A9	1	–	+	–	+	+	+	
A33	1	–	–	+	+	+	–	
A23	1	+	+	–	+	–	+	
A2a	1	–	–	+	+	–	+	
A35	1	–	–	+	+	–	–	
A26	1	–	–	–	+	+	+	
A19	1	+	–	–	+	–	–	
A2	1	+	–	–	–	–	–	
A17	1	–	–	–	+	–	–	
A87	1	+	+	–	NI	–	+	
A89	1	+	+	+	NI	+	+	
A75	1	+	+	+	+	+	+	*bla*_*OXA-51*_, *bla*_*OXA-23*_, *bla*_*VIM*_, IS*Aba1*, IS*Aba2*
A13a	1	–	+	+	+	–	–	
A40	1	+	+	+	+	+	–	*bla*_*OXA-51*_, *bla*_*OXA-23*_, *bla*_*VIM*_, *bla*_*NDM*_, IS*Aba1*
A59	1	+	+	+	+	–	+	
A85	1	+	+	–	+	+	+	
A11, A21, A70, A86	4	+	+	+	+	+	+	
A44	1	+	–	+	+	–	+	*bla*_*OXA-51*_, *bla*_*OXA-23*_, *bla*_*VIM*_, IS*Aba1*, IS*Aba3*
A77	1	+	+	+	+	+	+	*bla*_*OXA-51*_, *bla*_*OXA-23*_, *bla*_*VIM*_, *bla*_*NDM*_, IS*Aba1*, IS*Aba3*
A81	1	+	+	+	+	–	–	*bla*_*OXA-51*_, *bla*_*OXA-23*_, *bla*_*OXA-58*_, *bla*_*VIM*_, *bla*_*NDM*_, IS*Aba1*, IS*Aba3*

*MHT* Modified Hodge Test, *CIM* Carbapenem Inactivation Method, *CDT* Combined Disc Test, *NI* Non-interpretable

### Molecular detection of genes encoding different carbapenemases and insertion sequences

Out of the eleven genes investigated, six genes (*bla*_*KPC*_, *bla*_*IMP*_, *bla*_*SIM*_, *bla*_*SPM*_, *bla*_*GIM*_ and *bla*_*OXA-40*_) were not detected in any of the tested isolates. Two metallo-β-lactamases: *bla*_*VIM*_ and *bla*_*NDM*_ were detected in 74 (100%) and nine (12.1%) isolates, respectively. Sequencing revealed that all nine isolates carried *bla*_*NDM-1*_, the sequences were deposited in GenBank (accession numbers: MN395910, MN395911, MN395912, MN395913, MN395914, MN395915, MN395916, MN395917 and MN395918). Regarding the genes encoding class D carbapenemases, *bla*_*OXA-51*_ and *bla*_*OXA-23*_ were detected in all tested isolates (100%), while *bla*_*OXA-58*_ was detected in only one (1.4%) isolate: A81. On the other hand, insertion sequence IS*Aba1 was* detected in all tested isolates (100%), while IS*Aba2* and IS*Aba3* were detected in two (2.7%) and three isolates (4%), respectively (Table 3).

## Discussion

*A. baumannii* is becoming a major threat because of the dreadful number of nosocomial infections caused by this pathogen, mostly in ICUs worldwide [45, 46]. In addition, *A. baumannii* has become resistant to several antimicrobial classes due to the irrational use of antibiotics, leading to the predominance of multidrug-resistant strains particularly in hospital settings [47]. Moreover, carbapenem resistance among *A. baumannii* isolates restricts therapeutic options for treatment of such infections which might lead to higher morbidity and mortality rates [48, 49]. A few previous studies commented on the prevalence of carbapenemases among Egyptian *A. baumannii* clinical isolates [26, 27, 50–52].

Different mechanisms can contribute to carbapenem resistance, however, the production of MBL and CHDLs remain the most common and prevalent mechanisms among *A. baumannii* isolates [20]. MBLs are especially problematic because their genes are harbored on mobile elements, allowing their easy dissemination among the clinical isolates [49]. On the other hand, CHDLs can be either intrinsic/chromosomal or acquired β-lactamases [53]. Therefore, detection of carbapenemases among resistant strains is paramount to direct the proper treatment regimen.

This study aimed to phenotypically and molecularly characterize 74 Egyptian *A. baumannii* isolates to identify the different enzymes responsible for carbapenem resistance. Several phenotypic methods, including MHT, CIM, CDT, CarbAcineto NP test and boronic acid disc test were used. Phenotypic detection of carbapenemases has the advantages of low cost, ease of procedure and the absence of complicated or expensive equipment; however, it suffers from poor specificity and sensitivity. Therefore, PCR screening for some genes responsible for carbapenem resistance, as well as some insertion sequences was taken as the gold standard to evaluate the sensitivity of the different phenotypic methods.

Genes encoding for *bla*_*OXA-51*_ and *bla*_*OXA-23*_ belonging to class D carbapenemases were detected in 100% of the isolates, whereas *bla*_*OXA-58*_ was detected in only one isolate (A81) obtained in 2010. The *bla*_*OXA-51*_ is chromosomal and an intrinsic gene of *A. baumannii* species [30]. Among the acquired β-lactamases are OXA-23-like, OXA-24/40-like and OXA-58-like β-lactamases [53]. Numerous studies have lately reported that *bla*_*OXA-23*_ is the most prevalent carbapenemase gene identified among CR-AB isolates [53–56]. The prevalence rate of 100% reported in the current study is in agreement with previous studies [50, 54–57]. A recently published study characterizing 50 *A. baumannii* isolates from Mansoura, Egypt showed a *bla*_*OXA-51,*_ *bla*_*OXA-23*_ prevalence rates of 100 and 94%, respectively [58]. *bla*_*OXA-24/40*_ have mostly been found in the Iberian and Asian peninsulas and in other areas in the world [53–55, 59, 60]. Also, *bla*_*OXA-40*_ was detected in Egypt with a prevalence rate of 7.5% [51] and 2.9% [50]. However, in this study, *bla*_*OXA-40*_ was not detected in any of the tested isolates. There are many reports demonstrating the presence of *bla*_*OXA-58*_ in *A. baumannii* clinical isolates throughout different regions of the world including Algeria, Argentina, Kuwait, the UK, Italy, Turkey, the USA and Spain [57, 60–65]. The *bla*_*OXA-58*_ prevalence reported here (1.4%) is lower than previously published rates from Tunisia (4%), Egypt (9.1%) [50] and Algeria (14.7%) [61]. However, *bla*_*OXA-58*_ was absent in isolates collected in Mansoura, Egypt [58].

Of the genes encoding class B carbapenemases, *bla*_*VIM*_ and *bla*_*NDM*_ were detected in 100 and 12.1% of the isolates (9 out of 74), respectively. Three of the nine isolates were collected in 2015 showing a prevalence rate of 8.6% among the new collection whereas the remaining six isolates belonged to the older collection with a prevalence rate of 15.4%. More data are needed, preferably from different regions in the country, before we can safely conclude that the prevalence of *bla*_*NDM*_ is decreasing in Egypt. Benmahmod et al. [58] reported a *bla*_*VIM*_ and *bla*_*NDM*_ prevalence rates of 20 and 30%, respectively. The PCR screening results of *bla*_*NDM*_ were validated by sequencing. These results are in accordance with studies reported in China and Saudi Arabia [66–68]. Besides, *bla*_*NDM-1*_ and *bla*_*NDM-2*_ were reported in *A. baumannii* from Egypt [26, 27] and then disseminated in the entire Middle East [69]. None of the isolates were shown by PCR to carry *bla*_*KPC*_. Similar results were reported by Raible et al. [70], however Benmahmod et al. [58] reported a *bla*_*KPC*_ prevalence rate of 56%. Although *bla*_*SPM-1,*_ *bla*_*GIM,*_ *bla*_*SIM*_ and *bla*_*IMP*_ have been previously detected among Egyptian *A. baumannii* isolates [52, 56], none of the *A. baumannii* isolates in the current study harbored any of these genes.

Comparing the results of the phenotypic tests to the results of the molecular detection of carbapenemases showed that the sensitivity of MHT, CIM, CDT, CarbAcineto NP, boronic acid with imipenem and meropenem was 78.4, 68.9, 79.7, 95.9, 56.8 and 70.3%, respectively. In the CarbAcineto NP test, four isolates carrying *bla*_*OXA-51*,_ *bla*_*OXA-23*_, *bla*_*VIM,*_ including isolate no. A81 that additionally carried *bla*_*OXA-58*_ developed the positive result in less than 15 min which could be attributed to the activity of the enzymes in these isolates. The false negative result recorded with A2 that carried *bla*_*VIM*_, *bla*_*OXA-51*_ and *bla*_*OXA-23*_ could be explained by the low zinc concentration in the culture medium [71] or due to very low carbapenemase activity in the tested isolate [72]. These findings agree with the previously reported high sensitivity of CarbAcineto NP in carbapenemase detection among *Acinetobacter* spp. [39].

The sensitivity of MHT in the current study is also in agreement with previously published reports in which MHT was able to detect carbapenemase production in 83.3, 71 and 73% of the screened carbapenem-resistant isolates [73–75]. According to CLSI 2018, MHT is no longer recommended as a phenotypic test for carbapenemase detection, presumably because of the poor specificity of the test when detecting someextended spectrum β-lactamase production occurring with porin loss [76]. However, in the present study, all isolates shown by MHT to be carbapenemase producers also carried one or more carbapenemase genes as shown by PCR. The failure of CIM to detect carbapenemase production in 23 isolates could be due to the short incubation period of the meropenem disc relative to other studies that recommended six hours of incubation particularly with low level carbapenemase activity [77]. In the current study, CDT was capable of detecting the carbapenemases in 79.7% of the cases which is lower than the detection rate reported by Pandya et al. [78] (96.3%), Irfan et al. [79] (96.6%) and Anwar et al. [73] (95.4%). Boronic acid disc test has been reported to be an accurate phenotypic test for the detection of KPC carbapenemases [80–83]. However, the data concerning the application of the test for detection of other carbapenemases is unsatisfactory [84]. In the present work, no *bla*_*KPC*_ was detected.

Although only 21 isolates showed positive tests allover, all nine isolates that were shown to carry NDM by PCR also gave positive test in MHT, CIM and CarbAcineto NP making the sensitivity of these tests to detect MBL 100%. On the other hand, CDT failed to detect NDM in one isolate: A85 and boronic acid disc test with imipenem and meropenem failed to detect NDM in 2 isolates each: A59 and A81 and A40 and A81, respectively. It is noteworthy that A81 was the only isolate shown to carry *bla*_*OXA-58*_.

When present upstream to CHDL encoding genes, insertion sequences may increase the production of β-lactamases [65, 85]. In the current study, the prevalence of IS*Aba1*, IS*Aba2* and IS*Aba3* was 100, 2.7 and 4.1%, respectively. The prevalence of different insertion sequences in *A. baumannii* clinical isolates from Saudi Arabia was in agreement with the findings in the current study [68].

## Conclusions

With the exception of CarbAcineto NP that showed superior sensitivity approaching PCR results, a combination of phenotypic tests, including MHT, CIM, CDT and boronic acid disc tests seems essential for the conclusive detection of carbapenemases. NDM prevalence levels detected here are smaller than previously reported from other parts of the country which suggests the need for larger screening encompassing different Egyptian governorates to determine the exact prevalence rate. However, OXA-23 and VIM prevalence rates remain equally high.

## Data Availability

The datasets used and/or analyzed during the current study are available from the corresponding author on reasonable request.

## Abbreviations

ATCC: American Type Culture Collection
bp: Base pair
CDT: Combined disc test
CHDL: Carbapenem-hydrolyzing class D β-lactamases
CIM: Carbapenem inactivation method
CR-AB: Carbapenem-resistant *Acinetobacter baumannii*
DMSO: Dimethyl sulfoxide
DNA: Deoxyribonucleic acid
EDTA: Ethylene diamine tetraacetic acid
ESKAPE: *Enterococcus faecium*, *Staphylococcus aureus*, *Klebsiella pneumoniae*, *Acinetobacter baumannii*, *Pseudomonas aeruginosa* and *Enterobacter* species
ICU: Intensive care unit
IMP: Imipenemase
KPC: *Klebsiella pneumoniae* carbapenemase
LB: Luria-Bertani
MALDI-TOF MS: Matrix Assisted Laser Desorption Ionization - Time of Flight Mass Spectrometry
MBL: Metallo-β-lactamase
Mg/ml: Milligram per milliliter
MHT: Modified Hodge test
MIC: Minimum inhibitory concentration
Min: Minute
NDM: New Delhi metallo-β-lactamase
OXA: Oxacillinase
PBA: 3-aminophenylboronic acid
PCR: Polymerase chain reaction
Rpm: Round per minute
Sec: Second
SIM: Seoul Imipenemase
VIM: Verona integron-encoded metallo-β-lactamase
μg: Microgram
